# Association of Neighborhood Deprivation Index With Success in Cancer Care Crowdfunding

**DOI:** 10.1001/jamanetworkopen.2020.26946

**Published:** 2020-12-03

**Authors:** Elisabeth R. Silver, Han Q. Truong, Sassan Ostvar, Chin Hur, Nicholas P. Tatonetti

**Affiliations:** 1 Department of Medicine, Columbia University Irving Medical Center, New York, New York; 2 Herbert Irving Comprehensive Cancer Center, Columbia University Irving Medical Center, New York, New York; 3 Division of Digestive and Liver Diseases, Columbia University Irving Medical Center, New York, New York; 4 Department of Biomedical Informatics, Columbia University Irving Medical Center, New York, New York

## Abstract

**Importance:**

Financial toxicity resulting from cancer care poses a substantial public health concern, leading some patients to turn to online crowdfunding. However, the practice may exacerbate existing socioeconomic cancer disparities by privileging those with access to interpersonal wealth and digital media literacy.

**Objective:**

To test the hypotheses that higher county-level socioeconomic status and the presence (vs absence) of text indicators of beneficiary worth in campaign descriptions are associated with amount raised from cancer crowdfunding.

**Design, Setting, and Participants:**

This cross-sectional analysis examined US cancer crowdfunding campaigns conducted between 2010 and 2019 and data from the American Community Survey (2013-2017). Data analysis was performed from December 2019 to March 2020.

**Exposures:**

Neighborhood deprivation index of campaign location and campaign text features indicating the beneficiary’s worth.

**Main Outcomes and Measures:**

Amount of money raised.

**Results:**

This study analyzed 144 061 US cancer crowdfunding campaigns. Campaigns in counties with higher neighborhood deprivation raised less (–26.07%; 95% CI, –27.46% to –24.65%; *P* < .001) than those in counties with less neighborhood deprivation. Campaigns raised more funds when legitimizing details were provided, including clinical details about the cancer type (9.58%; 95% CI, 8.00% to 11.18%; *P* < .001) and treatment type (6.58%; 95% CI, 5.44% to 7.79%; *P* < .001) and financial details, such as insurance status (1.39%; 95% CI, 0.20% to 2.63%; *P* = .02) and out-of-pocket costs (7.36%; 95% CI, 6.18% to 8.55%; *P* < .001). Campaigns raised more money when beneficiaries were described as warm (13.80%; 95% CI, 12.30% to 15.26%; *P* < .001), brave (15.40%; 95% CI, 14.11% to 16.65%; *P* < .001), or self-reliant (5.23%; 95% CI, 3.77% to 6.72%; *P* < .001).

**Conclusions and Relevance:**

These findings suggest that cancer crowdfunding success ay disproportionately benefit those in high–socioeconomic status areas and those with the internet literacy necessary to portray beneficiaries as worthy. By rewarding those with existing socioeconomic advantage, cancer crowdfunding may perpetuate socioeconomic disparities in cancer care access. The findings also underscore the widespread nature of financial toxicity resulting from cancer care.

## Key Points

**Question:**

How is online crowdfunding for cancer care associated with existing socioeconomic health disparities in the US cancer care setting?

**Findings:**

In this cross-sectional study of 144 061 cancer crowdfunding campaigns, those located in US counties with high socioeconomic status raised significantly more than campaigns in lower–socioeconomic status counties. Crowdfunders who used campaign narratives to portray beneficiaries as worthy of donations raised significantly more than those without such portrayals, and the use of these portrayals was unequally distributed across socioeconomic strata.

**Meaning:**

These findings suggest that crowdfunding’s reliance on access to interpersonal wealth and proficiency in digital self-marketing may disproportionately benefit those with existing socioeconomic advantage.

## Teaser

This cross-sectional study examines whether higher county-level socioeconomic status and the presence (vs absence) of text indicators of beneficiary worth in campaign descriptions are associated with the amount raised from cancer crowdfunding.

## Introduction

US individuals experience high rates of disease-related financial burden, with 1 in 4 reporting trouble paying medical bills.^1^ Rates of financial distress due to medical expenses among patients with cancer in particular are estimated to range from 16% to 73%.^2,3^ Treatment-related financial hardship can lead patients to adopt unsustainable coping methods, including debt accumulation, medication nonadherence, foregoing medical appointments, and exhausting savings.^4,5^ Thus, it is not surprising that financial toxicity (FT) resulting from cancer care is associated with adverse mental and physical health outcomes.^2,6,7^ To alleviate FT, some patients with cancer have turned to online fundraising, or crowdfunding.

Crowdfunding campaigns solicit donations from friends, family, and strangers, mediated by websites that host pages describing the beneficiary’s story and needs. Effective campaigns successfully engage more donors by relaying a sympathetic narrative of the recipient,^8^ thus demonstrating the beneficiary’s worth or deservingness for financial assistance. Not surprisingly, past work on medical crowdfunding has raised the concern that crowdfunding may exacerbate existing socioeconomic disparities by disproportionately benefiting those with access to interpersonal wealth and the internet, as well as those with the digital media literacy necessary to successfully frame an online campaign.^9,10,11,12,13^ This concern is especially poignant given the positive association between internet literacy and socioeconomic status (SES) among children,^14^ college students,^15^ and adults.^16^

In the context of crowdfunding, internet and digital literacy may be understood as proficiency in presenting a beneficiary as worthy of donations by appealing to perceptions of deservingness. The stereotype content model is a framework for studying social judgments along 2 dimensions of perceived warmth and competence.^17^ Applied to SES, low-SES individuals are stereotyped as warmer but less competent than high-SES individuals.^18^ Consequently, emphasizing a beneficiary’s warmth, gratitude, and kindness can communicate their worth.^19^ Crowdfunders may also benefit by avoiding language that could lead donors to perceive the beneficiary as incompetent and responsible for their hardship or as misleading donors regarding the true extent of their needs.^18,20,21,22,23^ Specific to cancer, language describing an individual’s compliance with treatment by battling their cancer while remaining brave may also position a beneficiary as more deserving of donations.^23^

Quantitative studies have yet to examine the associations among SES, text indicators of worth, and cancer crowdfunding on a large scale within the US cancer care setting. To address this gap in the literature, we leveraged open-source data mining tools to analyze all US cancer crowdfunding campaigns shared publicly on GoFundMe.com. Given past work highlighting the importance of digital literacy in perpetuating cancer care disparities through crowdfunding,^8,9,11,12^ we focused on crowdfunders’ ability to portray beneficiaries as deserving and sympathetic, in addition to the geographical socioeconomic context of the campaign. We tested the hypotheses that the amount raised by campaigns, but not goal amount, would be associated with higher county-level SES and text describing the beneficiary’s worth, including positive stereotypes of low-SES individuals, distancing from negative stereotypes of low-SES individuals, legitimizing clinical and financial details, and consistency with cancer narratives. We also posited that campaigns in higher-SES counties would more often use the text features describing the beneficiary’s worth.

## Methods

This cross-sectional study used public crowdfunding data from GoFundMe.com and InternetArchive.org, socioeconomic data from the US Census Bureau, and geographic information from Mapbox^24,25^ and the Federal Communications Commission. Columbia University Medical Center’s institutional review board determined this research to be exempt and classified it as non–human subjects research; thus, informed consent was not needed, in accordance with 45 CFR §46. The code used for this analysis is available on Github.^26^ This report follows the Strengthening the Reporting of Observational Studies in Epidemiology (STROBE) reporting guideline for cross-sectional studies.

### Online Fundraising Data Collection and Reduction

We implemented a web crawler in Python programming language version 3.7 (Python) to automatically retrieve information from individual campaign pages using GoFundMe.com’s public sitemap.^27^ To account for missing information and old campaigns taken down before our collection date, we deployed a second web crawler to scrape archived GoFundMe.com webpages available through InternetArchive.org’s Wayback Machine application programming interface (API).^28^ We conducted this search on September 10, 2019, and scraped 1 856 154 pages in total. For each campaign, we collected the campaign title, creation date, location, description, tag for campaign category, amount of money raised, goal amount, number of contributors, number of social media shares, and number of likes or followers.

Next, we curated a cancer-specific subset of the resulting data set (Table 1), yielding 144 752 cancer crowdfunding campaigns. Campaigns were selected on the basis of a keyword search of titles and descriptions (eAppendix 1 in the Supplement). Because we intended to capture campaigns for individuals (rather than organizations or initiatives), we restricted campaigns to only those categorized as medical by users in GoFundMe.com’s mutually exclusive campaign categories.

**Table 1.** Descriptive Statistics

Sample characteristics	Count
Total URLs scraped	1 856 154
Excluded	
Duplicate campaigns	53 296
Low-quality data	26 451
Non-US location or currency	249 981
Noncancer campaigns (based on keyword search)	1 304 279
Tag categories other than medical	77 154
Campaigns missing year	151
Campaigns that failed geocoding	542
Campaigns with *research* in campaign title	149
Final sample	144 061
Campaign characteristics, mean (SD)	
Amount raised, $	6367.77 (11 611.49)
Goal amount, $	18 123.95 (21 912.28)
Contributors, No.	64.05 (129.89)
Mean donation amount, $	102.98 (101.58)
Percentage of goal raised	43.99 (32.19)
Social media shares, No.	436.71 (863.09)
Met fundraising goal, No. (%)	15 760 (10.94)
Neighborhood deprivation index quartile	
1 (Least deprived)	53 252 (36.96)
2	42 769 (29.69)
3	35 613 (24.72)
4 (Most deprived)	12 427 (8.63)
Year, mean (SD), no. of campaigns	
2010	13 (0.01)
2011	115 (0.08)
2012	682 (0.47)
2013	2874 (1.99)
2014	10 368 (7.20)
2015	19 916 (13.82)
2016	18 082 (12.55)
2017	30 256 (21.00)
2018	38 983 (27.06)
2019	22 772 (15.81)
Text mentions	
Warmth	26 161 (18.16)
Gratitude	103 206 (71.64)
Self-reliance	21 378 (14.84)
Cancer type	119 976 (83.28)
Treatment type	97 126 (67.42)
Insurance	40 428 (28.06)
Out-of-pocket cost	58 543 (40.64)
Bravery	41 409 (28.74)
Militaristic metaphors	82 543 (57.30)

Using location names provided by the campaign posters, we geocoded these 144 752 campaigns using a geocoding API from Mapbox.^24,25^ We then used an API from the Federal Communications Commission to map the latitudes and longitudes to county federal information processing codes,^29^ allowing for linkage with county-level SES US Census data. Campaigns failed to geocode if the location was indecipherable (eg, a non–zip code number) or was not in the US. In total, 542 campaigns failed to geocode. We then excluded any campaigns with *research* in the title to reduce the risk of including campaigns not intended for individuals with cancer. This resulted in a final data set of 144 061 cancer crowdfunding campaigns between the years 2010 and 2019 (Table 1).

After curating the final data set, we Winsorized the goal amount to handle extreme outliers by reassigning values below the 5th percentile and above the 95th percentile, as recommended by past work.^30^ Using this method, 6.32% of values (9106 campaigns) were reassigned, such that values above the 95th percentile (2113 campaigns [1.47%]) were reassigned to $100 000 (the 95th percentile value), and values below the 5th percentile (6993 campaigns [4.85%]) were reassigned to $2000 (the 5th percentile value).

### Exposures and Outcome

#### Socioeconomic Data

We extracted a number of socioeconomic variables^31,32,33^ from the American Community Survey using the US Census Bureau’s API (see eAppendix 2 and eFigure 1 in the Supplement for details).^34^ We used 5-year estimates (2013-2017) for all variables and performed a principal components analysis in 2 steps to compute a single neighborhood deprivation index (NDI), similar to past work.^31,32,33^

After running the initial principal components analysis, we omitted variables with a factor loading less than 0.25.^31,33^ On the basis of this criterion, the final NDI included unemployment, poverty, percentage uninsured, high school completion, internet access, households headed by a single parent, and households with annual income less than $35 000. These factors explained 59.25% of the variability across counties, similar to past work,^33,35^ and we used them to calculate standardized NDIs (see eAppendix 2 in the Supplement for factor loadings and details). NDI scores were grouped into quartiles,^31,32,33^ and index scores were matched to campaigns according to the campaign’s county federal information processing code.

#### Text Mining

To extract features related to self-reliance, militaristic metaphors, bravery, clinical details, and financial details, we conducted a series of keyword searches using regular expressions, as detailed in eTable 1, eTable 2, eTable 3, and eAppendix 3 in the Supplement. Each search was intended to capture both a single key word (eg, *brave*) and words indicating similar constructs (eg, *courage*, *strength*, and *hero*). Campaigns were categorized as either containing or not containing the construct in question according to the results of the keyword search.

#### Campaign Fundraising

Our primary outcome was the amount raised by crowdfunding campaigns. We also examined campaign goal amount as a secondary campaign outcome.

### Statistical Analyses

#### Univariable Analyses

We generated descriptive statistics to characterize the monetary campaign information, NDI quartile distributions, data missingness, and presence of text features. For all analyses, we omitted cases with missing data for the variable of interest. To test the hypothesis that the amount raised by campaigns, but not goal amount, would be associated with higher county-level SES, we used 1-way analyses of variance comparing mean amount raised and goal amount across the 4 NDI quartiles. All tests for significance were 2-sided. We examined significant (*P* < .05) overall differences by NDI quartile using post hoc Tukey HSD tests. To test the hypothesis that the amount raised would be associated with text describing the beneficiary’s worth, we used independent samples *t* tests assessing differences in amount raised and goal amount by presence of the following text features: warmth, gratitude, self-reliance, cancer type, treatment type, insurance, out-of-pocket costs, bravery, and militaristic metaphors. We used χ^2^ tests of independence to compare the observed and expected counts of each text mention listed across NDI quartile. We probed significant overall associations using pairwise χ^2^ tests with a Bonferroni correction for the 6 pairwise comparisons between NDI quartiles, resulting in a significance threshold of *P* < .0083.^36^

#### Multivariable Analysis

We constructed a generalized linear regression model to estimate associations between amount raised with campaign textual characteristics and SES, while adjusting for variation attributable to social media shares, goal amount, year of creation, and number of campaign contributors. We selected these variables a priori on the basis of past work^37^ and author assumptions. We confirmed their inclusion in the model as potential confounders on the basis of significant univariable associations with amount raised (eTable 3 and eTable 4 in the Supplement). To account for nonnormality in the distribution of errors and considerable positive skew in amount raised (eTable 5 in the Supplement), we log-transformed the amount raised and entered it as the dependent variable in regression models. We calculated the expected percentage change in amount raised when a given feature was present.^38^ Data analysis was performed using Python version 3.7 and R statistical software version 3.6.3 (R Project for Statistical Computing) from December 2019 to March 2020.

## Results

### Univariable Analyses

We curated the largest reported sample of US cancer crowdfunding campaigns to date, with 144 061 campaigns included in the analyses. Descriptive statistics are displayed in Table 1 and eTable 5 in the Supplement. An analysis of variance indicated significant differences in amount raised across NDI quartiles. The mean (SD) amounts raised were $7402.94 ($12 184.61) for NDI 1 (the least deprived quartile), $6009.28 ($10 740.33) for NDI 2, $5775.41 ($12 183.32) for NDI 3, and $4857.87 ($9797.49) for NDI 4 (the most deprived quartile) (*F*_3,143 568_ = 256.00; *P* < .001) (Table 2). Tukey honest significant different post hoc tests revealed a dose-dependent effect, such that the amount raised increased significantly with decreasing levels of deprivation. However, there was also a significant difference in the stated goal amount by NDI quartile. As with amount raised, those in less deprived counties sought a higher goal amount than those in more deprived counties, although the means for counties in the second and third deprivation quartiles did not significantly differ from one another (see eFigure 2 in the Supplement).

**Table 2.** Amount Raised, Goal Amount, and Text Feature Usage by NDI Quartile

Variable	NDI quartile				Test statistic	*P* value^a^
	1 (Least deprived) (n = 53 252)	2 (n = 42 769)	3 (n = 35 613)	4 (Most deprived) (n = 12 427)		
Amount raised, mean (SD), $	7402.94 (12 184.61)	6009.28 (10 740.33)	5775.41 (12 183.32)	4857.87 (9797.49)	*F*_3,143 568_ = 256.00	<.001
Goal amount, mean (SD), $	19 000.14 (22 188.29)	17 693.08 (21 739.73)	17 941.10 (21 926.82)	16 363.95 (21 092.59)	*F*_3,140 231_ = 59.89	<.001
Text mentions, % (N = 144 061)^b^						
Warmth	19.73	18.10	17.06	14.79	χ^2^_3_ = 212.75	<.001
Gratitude	72.12	71.48	71.43	70.70	χ^2^_3_ = 12.76	.005
Self-reliance	15.79	14.92	14.16	12.39	χ^2^_3_ = 110.46	<.001
Cancer type	83.90	83.51	82.69	81.50	χ^2^_3_ = 53.62	<.001
Treatment type	68.21	67.49	67.19	64.44	χ^2^_3_ = 66.43	<.001
Insurance	27.56	28.20	28.61	28.16	χ^2^_3_ = 12.33	.006
Out-of-pocket cost	41.81	40.96	39.72	37.13	χ^2^_3_ = 108.06	<.001
Militaristic metaphors	58.38	57.01	57.03	54.43	χ^2^_3_ = 69.64	<.001
Bravery	30.23	28.53	27.83	25.74	χ^2^_3_ = 127.25	<.001

Abbreviation: NDI, neighborhood deprivation index.

^a^ Significance was set at *P* < .0083.

^b^ Percentages are within NDI group.

As shown in Table 3, campaigns that mentioned a beneficiary’s warmth or gratitude raised significantly more money than those that did not (mean [SD], $8050.41 [$13 196.40] vs $5993.32 [$11 194.11]; *t*_143 572_ = 25.96; *P* < .001), but they also indicated a significantly higher goal amount. Campaigns that described the beneficiary as self-reliant raised significantly more than those that did not (mean [SD], $7515.91 [$13 398.57] vs $6167.24 [$11 258.40]; *t*_143 572_ = 15.67; *P* < .001) but had a significantly higher goal amount. Those who mentioned a specific cancer type, specific treatment, insurance type, or out-of-pocket costs raised significantly more money than campaigns that did not mention these features, although they also requested a higher goal amount. Finally, campaigns that invoked militaristic metaphors or described the beneficiary as brave raised significantly more money than campaigns that did not, but they also requested a higher goal amount than campaigns without these features (Table 3).

**Table 3.** Amount Raised and Goal Amount by Campaign Text Characteristics

Amounts raised and goal amounts by text feature	Mean (SD), $		*t* Statistic^a^
	Feature is present	Feature is not present	
Amount raised			
Warmth	8050.41 (13 196.40)	5993.32 (11 194.11)	*t*_143 572_ = 25.96
Gratitude	6574.33 (11 929.26)	5844.35 (10 746.74)	*t*_143 572_ = 10.73
Self-reliance	7515.91 (13 398.57)	6167.24 (11 258.40)	*t*_143 572_ = 15.67
Cancer type	6628.37 (11 519.45)	5063.79 (11 976.87)	*t*_143 572_ = 19.05
Treatment type	6692.74 (11 616.68)	5692.98 (11 571.71)	*t*_143 572_ = 15.29
Insurance	7036.93 (13 392.90)	6106.43 (10 825.39)	*t*_143 572_ = 13.65
Out-of-pocket costs	6886.10 (11 952.27)	6012.10 (11 358.14)	*t*_143 572_ = 14.02
Militaristic metaphors	7107.79 (12 608.71)	5371.39 (10 028.91)	*t*_143 572_ = 28.10
Bravery	7950.95 (13 078.49)	5727.55 (10 897.60)	*t*_143 572_ = 32.98
Goal amount			
Warmth	21 472.19 (23 808.50)	17 367.20 (21 388.12)	*t*_140 235_ = 27.27
Gratitude	18 369.91 (21 969.53)	17 468.45 (21 745.66)	*t*_140 235_ = 6.86
Self-reliance	20 370.80 (23 120.85)	17 724.98 (21 666.39)	*t*_140 235_ = 16.20
Cancer type	18 719.85 (22 220.94)	14 930.15 (19 875.96)	*t*_140 235_ = 23.62
Treatment type	18 734.16 (22 117.25)	16 800.62 (21 401.59)	*t*_140 235_ = 15.37
Insurance	21 378.90 (24 134.97)	16 827.97 (20 820.77)	*t*_140 235_ = 35.26
Out-of-pocket costs	19 732.85 (22 579.00)	16 992.59 (21 358.72)	*t*_140 235_ = 23.10
Militaristic metaphors	19 683.29 (22 813.04)	16 006.36 (20 436.72)	*t*_140 235_ = 31.16
Bravery	20 617.33 (23 354.92)	17 096.58 (21 204.44)	*t*_140 235_ = 27.43

^a^ *P* < .001 for all comparisons.

We found that the use of text features indicating deservingness would be unequally distributed across NDI quartiles. In general, campaigns in the least deprived quartile used text features indicating deservingness more often than those in more deprived quartiles, with the exception of insurance, which was mentioned more often in campaigns in more deprived quartiles (see Table 2 for distributions and pairwise comparisons and eTable 6 in the Supplement for expected and observed counts).

### Multivariable Analysis

The results of a generalized linear regression model with amount raised as the dependent variable are summarized in Table 4 and the Figure. After adjusting for potential effects of social media shares, campaign year, number of contributors, and goal amount on amount raised, we found results similar to those reported in univariable analyses (see eTable 7 in the Supplement for full regression model outputs). Campaigns in the most deprived NDI quartile group raised 26.07% (95% CI, –27.46% to –24.65%; *P* < .001) less than those in the least deprived NDI quartile group, those in the third NDI quartile raised 18.14% less than those in the least deprived quartile, and those in the second NDI quartile raised 13.39% less than those in the least deprived quartile. Campaigns that mentioned a beneficiary’s warmth raised significantly more than those that did not (13.80%; 95% CI, 12.30% to 15.26%; *P* < .001), but the association between amount raised and gratitude was not significant, as did campaigns that mentioned the beneficiary’s self-reliance (5.23%; 95% CI, 3.77% to 6.72%; *P* < .001) and bravery (15.40%; 95% CI, 14.11% to 16.65%; *P* < .001). We found that greater detail regarding the beneficiary’s need were associated with a greater amount raised. Campaigns that mentioned the beneficiary’s cancer type (9.58%; 95% CI, 8.00% to 11.18%; *P* < .001), treatment type (6.58%; 95% CI, 5.44% to 7.79%; *P* < .001), insurance (1.39%; 95% CI, 0.20% to 2.63%; *P* = .02), or out-of-pocket costs (7.36%; 95% CI, 6.18% to 8.55%; *P* < .001) raised significantly more than campaigns that did not mention these needs (Table 4). Finally, campaigns that mentioned the beneficiary’s bravery or used militaristic metaphors raised significantly more money than campaigns without these features.

**Table 4.** Results of Multivariable Regression Model for Amount Raised

Variable	β coefficient (SE)	Difference in expected, mean (95% CI), %^a^	*P* value
NDI quartile 2 (reference: NDI quartile 1, least deprived)	–0.144 (0.006)	–13.39 (–14.44 to –12.28)	<.001
NDI quartile 3 (reference: NDI quartile 1, least deprived)	–0.200 (0.007)	–18.14 (–19.18 to –17.06)	<.001
NDI quartile 4 (most deprived) (reference: NDI quartile 1, least deprived)	–0.302 (0.010)	–26.07 (–27.46 to –24.65)	<.001
Warmth is mentioned (reference: warmth not mentioned)	0.129 (0.007)	13.80 (12.30 to 15.26)	<.001
Gratitude is mentioned (reference: gratitude not mentioned)	0.001 (0.006)	0.09 (–1.00 to 1.21)	.88
Self-reliance is mentioned (reference: self-reliance not mentioned)	0.051 (0.007)	5.23 (3.77 to 6.72)	<.001
Cancer type is mentioned (reference: cancer type not mentioned)	0.091 (0.007)	9.58 (8.00 to 11.18)	<.001
Treatment type is mentioned (reference: treatment type not mentioned)	0.064 (0.006)	6.58 (5.44 to 7.79)	<.001
Insurance is mentioned (reference: insurance not mentioned)	0.014 (0.006)	1.39 (0.20 to 2.63)	.02
Out-of-pocket cost is mentioned (reference: out-of-pocket cost not mentioned)	0.071 (0.005)	7.36 (6.18 to 8.55)	<.001
Militaristic metaphors (reference: militaristic metaphors not used)	0.112 (0.005)	11.87 (10.74 to 13.09)	<.001
Bravery is mentioned (reference: bravery not mentioned)	0.143 (0.006)	15.40 (14.11 to 16.65)	<.001

Abbreviation: NDI, neighborhood deprivation index.

^a^ Full model outputs are detailed in eTable 7 in the Supplement.

**Figure.**
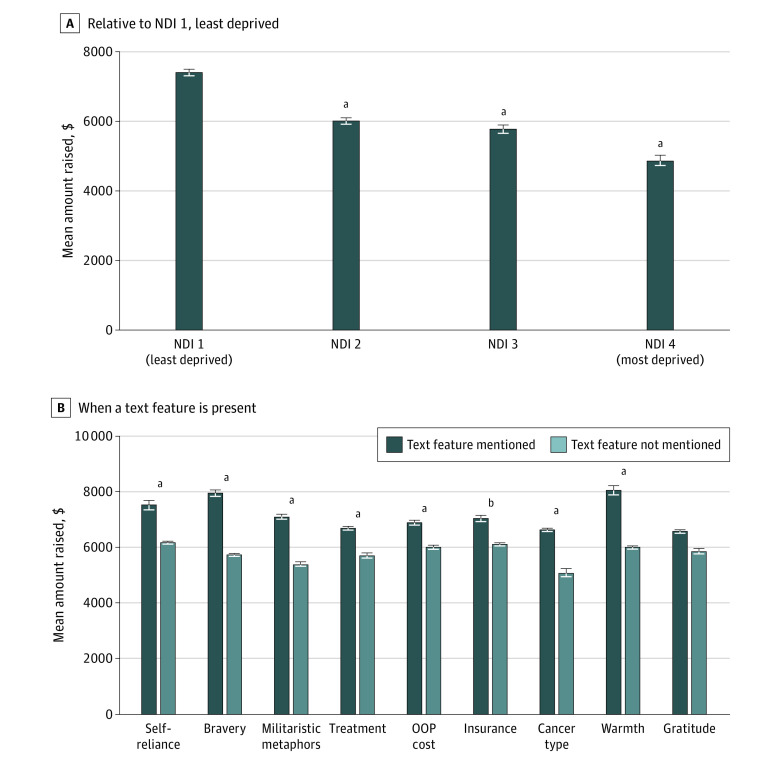
Results of Multivariable Regression Model for Amount Raised A, Bars represent mean amount of money raised in campaigns in each neighborhood deprivation index (NDI) level, with error bars denoting bootstrapped 95% CIs. Compared with the least deprived quartile (NDI 1), campaigns in NDI 2 areas raised 13.39% less, those in NDI 3 areas raised 18.14% less, and those in NDI 4 areas raised 26.07% less. B, Bars indicate mean amount of money raised when a particular text feature is present or absent, according to the fitted multivariable regression model described in Table 4. The differences in amounts raised between campaigns that did or did not mention the text feature were 5.23% for self-reliance, 15.40% for bravery, 11.87% for militaristic metaphors, 6.58% for type of treatment, 7.36% for out-of-pocket (OOP) costs, 1.39% for insurance, 9.58% for cancer type, 13.80% for warmth, and 0.09% for gratitude. ^a^*P* < .001. ^b^*P* < .05.

## Discussion

This cross-sectional analysis found that crowdfunding campaigns for cancer care expenses raised significantly more money when they were located in US counties with higher SES and when they described beneficiaries as worthy of donations (eg, as consistent with positive low-SES stereotypes, inconsistent with negative low-SES stereotypes, or aligned with cancer narratives of bravery and battle imagery).^39,40^ The use of these text features was unequally distributed across socioeconomic strata: campaigns in higher-SES counties tended to use these indicators more often than those in lower-SES counties. These findings suggest that online crowdfunding may exacerbate socioeconomic disparities in cancer care and also highlight the widespread nature of difficulty paying for cancer care. As cancer care costs continue to increase,^41,42,43^ FT resulting from cancer care poses a significant public health issue. Although lower-SES patients are at greater risk of FT, we found that these patients are the least likely to benefit from cancer crowdfunding as a way to mitigate it.

Although qualitative and theoretical work on medical crowdfunding has suggested that the practice may perpetuate socioeconomic health disparities,^9,11,12,44^ quantitative work on cancer crowdfunding is limited. One Canadian study of 1788 campaigns linked geospatial campaign counts with corresponding socioeconomic data and found a positive association between crowdfunding use and SES.^13^ Our study confirms their findings in a much larger sample of 144 061 US cancer crowdfunding campaigns. In addition to corroborating previous associations between high SES and cancer crowdfunding, our results demonstrate the importance of campaign text characteristics, particularly characteristics that portray beneficiaries as worthy. As with other indicators of digital and internet literacy,^15,16,45,46^ the use of personal marketing strategies was unevenly distributed across socioeconomic strata, often to the benefit of those in higher-SES areas. This supports previously noted concerns that medical crowdfunding disproportionately benefits those with the requisite internet literacy.^9,11,12,44^

Our findings are comparable to recent work by Cohen and colleagues^47^ focusing on explicit mentions of being insured and uninsured in US cancer crowdfunding campaigns. In their sample of 1035 campaigns, Cohen et al^47^ found no differences in amount raised by insurance status but did find higher goal amounts among uninsured and underinsured beneficiaries. We found similar results for goal amount, but we also found a small but significant association between mentioning insurance and amount raised. This discrepancy may be due to our broader definition of insurance mention but may also be related to our novel approach to web scraping and data reduction. Although previous studies have used GoFundMe.com’s internal search bar to find cancer crowdfunding campaigns,^13,37,47^ we leveraged the domain’s sitemap to collect all indexed campaigns. Because internal search bars commonly use search engine optimization to return results with greater engagement potential and more recent results, our method decreased the risk of this bias by collecting URLs directly from GoFundMe.com’s sitemap.

### Limitations

Although, to our knowledge, our study offers the largest and most comprehensive quantitative analysis of cancer crowdfunding campaigns in the US to date, it is limited in some respects. First, using automated text mining rather than manual coding may include campaigns in counts of text features when the use context of certain words does not align with the connotation we assumed. Similarly, we were unable to extract information regarding cancer stage at campaign initiation. A related concern is that crowdfunding campaigns are often initiated by someone other than the beneficiaries themselves. Future work would do well to use natural language processing techniques to explore this issue. Our automated approach to geocoding the data set carries similar limitations related to trade-offs between precision and quantity. Because geocoding relied on user-provided locations with varying levels of granularity, the smallest unit of geographic analysis for US Census data was the county. Further insights may be gleaned by collaborating with crowdfunding platforms to obtain primary data. Because we did not have access to primary data such as the campaign initiator’s internet provider address, we were not able to compare the user-defined campaign location with the initiator’s location. Despite our novel approach to data collection, our sample is prone to biases in data availability. Earlier campaigns may be sparse because of users deleting campaigns in the time since initiation. Because campaigns can be listed as inactive for any reason, it is difficult to anticipate how these campaigns would influence results. We were able to recover data for some inactive campaigns by scraping archived GoFundMe.com webpages with the InternetArchive.org’s API. Future research regarding campaign closure, including time from initiation to closure and reasons for closure, may provide additional insights. Furthermore, we did not collect other data from campaigns that may be informative, including the number of campaign updates, campaign comments from other users, and image data.

## Conclusions

To our knowledge, this cross-sectional study is the first to provide large-scale quantitative support for the notion that cancer crowdfunding may perpetuate SES disparities in US cancer care. In particular, we found that crowdfunders with the digital literacy necessary to market beneficiaries as worthy and those in higher-SES areas raised significantly more money through online cancer crowdfunding than those without these advantages. Our results suggest that rather than remedying socioeconomic disparities in cancer care, online crowdfunding provides additional privileges to those with existing socioeconomic advantage, potentially exacerbating SES disparities and further marginalizing those most at risk of FT.

## Notes

Supplement. **eAppendix 1.** Search Terms Used to Determine Cancer Campaigns **eAppendix 2.** Supplemental Methods: Neighborhood Deprivation Index **eFigure 1.** US Census Socioeconomic Data by Neighborhood Deprivation Index Quartile **eTable 1.** Search Terms Used to Determine and Recode Cancer Type **eTable 2.** Search Terms and Regular Expressions (Regex) Used to Determine and Recode Mentions of Insurance, Out-of-Pocket Costs, and Treatment Type **eAppendix 3.** Keyword Searches for Deservingness Text Features **eTable 3.** Associations Between Amount Raised and Campaign Year **eTable 4.** Spearman Rank Correlations Between Amount Raised and Relevant Continuous Variables **eTable 5.** Descriptive Statistics for Continuous Variables **eFigure 2.** Log Amount Raised by Neighborhood Deprivation Index **eTable 6.** Expected and Observed Counts for Text Indicators by County Socioeconomic Status **eTable 7.** Full Outputs From Multivariable Regression Model on Amount Raised (Log-Transformed) **eReferences**
