# Supplementary material for: Association of Neighborhood Deprivation Index With Success in Cancer Care Crowdfunding
Source: JAMA Netw Open. 2020 Dec 3;3(12):e2026946. doi: 10.1001/jamanetworkopen.2020.26946 (PMC7716189; doi:10.1001/jamanetworkopen.2020.26946)
Supplement: Supplement. — eAppendix 1. Search Terms Used to Determine Cancer Campaigns eAppendix 2. Supplemental Methods: Neighborhood Deprivation Index eFigure 1. US Census Socioeconomic Data by Neighborhood Deprivation Index Quartile eTable 1. Search Terms Used to Determine and Recode Cancer Type eTable 2. Search Terms and Regular Expressions (Regex) Used to Determine and Recode Mentions of Insurance, Out-of-Pocket Costs, and Treatment Type eAppendix 3. Keyword Searches for Deservingness Text Features eTable 3. Associations Between Amount Raised and Campaign Year eTable 4. Spearman Rank Correlations Between Amount Raised and Relevant Continuous Variables eTable 5. Descriptive Statistics for Continuous Variables eFigure 2. Log Amount Raised by Neighborhood Deprivation Index eTable 6. Expected and Observed Counts for Text Indicators by County Socioeconomic Status eTable 7. Full Outputs From Multivariable Regression Model on Amount Raised (Log-Transformed) eReferences [file jamanetwopen-e2026946-s001.pdf]

Supplemental Online Content

Silver ER, Truong HQ, Ostvar S, Hur C, Tatonetti NP. Association of neighborhood deprivation index with success in cancer care crowdfunding. *JAMA Netw Open*. 2020;3(12):e2026946. doi:10.1001/jamanetworkopen.2020.26946

eAppendix 1. Search Terms Used to Determine Cancer Campaigns

eAppendix 2. Supplemental Methods: Neighborhood Deprivation Index

eFigure 1. US Census Socioeconomic Data by Neighborhood Deprivation Index Quartile

eTable 1. Search Terms Used to Determine and Recode Cancer Type

eTable 2. Search Terms and Regular Expressions (Regex) Used to Determine and Recode Mentions of Insurance, Out-of-Pocket Costs, and Treatment Type

eAppendix 3. Keyword Searches for Deservingness Text Features

eTable 3. Associations Between Amount Raised and Campaign Year

eTable 4. Spearman Rank Correlations Between Amount Raised and Relevant Continuous Variables

eTable 5. Descriptive Statistics for Continuous Variables

eFigure 2. Log Amount Raised by Neighborhood Deprivation Index

eTable 6. Expected and Observed Counts for Text Indicators by County Socioeconomic Status

eTable 7. Full Outputs From Multivariable Regression Model on Amount Raised (Log-Transformed)

eReferences

This supplemental material has been provided by the authors to give readers additional information about their work.

© 2020 Silver ER et al. *JAMA Network Open.*

eAppendix 1. Search Terms Used to Determine Cancer Campaigns. Search terms were sed to subset cancer campaigns from all campaigns.

- malignan
- carcinoma
- cancer
- radiation therap
- radiotherap
- immuno therap
- immunotherap
- chemo
- chemotherap
- lymphoma
- sarcoma
- melanoma
- glioblastoma
- myeloma
- neuroblastoma
- astrocytoma
- renal cell
- squamous cell
- medulloblastoma
- adenocarcinoma
- retinoblastoma
- ductal carcinoma
- mastectomy
- neuroendocrine tumor
- histiocytosis
- luekemia
- carcinoid
- lukemia
- germ cell tumor
- desmoplastic
- wilms tumor
- seminoma
- ependymoma
- thymoma
- nueroblastoma
- lumpectomy
- myloma
- langerhans
- ductile carcinoma
- oligodendroglioma
- wilm's tumor
- clear cell

© 2020 Silver ER et al. *JAMA Network Open.*

- non-hodgkins lymphoma
- non hodgkins lymphoma
- neurblastoma
- neruoblastoma
- rhabdomyosaroma
- nuroblastoma
- nonhodgkins lymphoma
- rhabdomyosarcoma

© 2020 Silver ER et al. *JAMA Network Open.*

eAppendix 2. Supplemental Methods: Neighborhood Deprivation Index. In the first step of the Principal Components Analysis (PCA) to calculate neighborhood deprivation index (NDI) quartiles, we included the following standardized county-level variables (proportions of county populations):

- Unemployment
- Households without a car
- Crowding (households with more than 1 person occupying each room)
- Vacant housing units
- High school completion
- Percent Black or African-American
- Home ownership
- Households headed by a single parent
- Households with annual income < $35,000
- Poverty
- Households receiving public assistance
- Employment in management, the arts, or science
- Percent without health insurance
- Internet access

The PCA was then run a second time, omitting variables with factor loadings less than 0.25.[1,2] Factor loadings were transformed by multiplying each result by -1 so that higher NDI metrics indicated greater deprivation. Factor loadings served as weights to compute county-level neighborhood deprivation indices (NDIs) based on the county values of the corresponding standardized Census variables. For analysis, we normalized NDIs and grouped the counties into NDI quartiles, with lower quartiles representing less deprivation and higher quartiles representing greater deprivation. NDI quartiles and index scores were matched to campaigns based on the campaign's county FIP code.

**Variable**	**Loading: Full PCA**	**Loading: Reduced PCA**
Unemployment Rate	0.31	0.34
Poverty Rate	0.39	0.45
% without Health Insurance	0.27	0.32
Home Ownership Rate	-0.15	NA
% with >1 Person per Room	0.21	NA
% without a Car	0.24	NA
% Vacant Units	0.08	NA
% with High School Education	-0.34	-0.4
% with Internet Access	-0.31	-0.39
% African American	0.23	NA
% Single Parent	0.28	0.29

© 2020 Silver ER et al. *JAMA Network Open.*

% with Annual Income < $35,000	0.34	0.42
% on Public Assistance	0.16	NA
% in Management, Arts, and Science Occupations	-0.24	NA
**Variance Explained**	**38.75%**	**59.25%**

© 2020 Silver ER et al. *JAMA Network Open.*

eFigure 1. US Census Socioeconomic Data by Neighborhood Deprivation Index Quartile.
NDI: Neighborhood Deprivation Index.

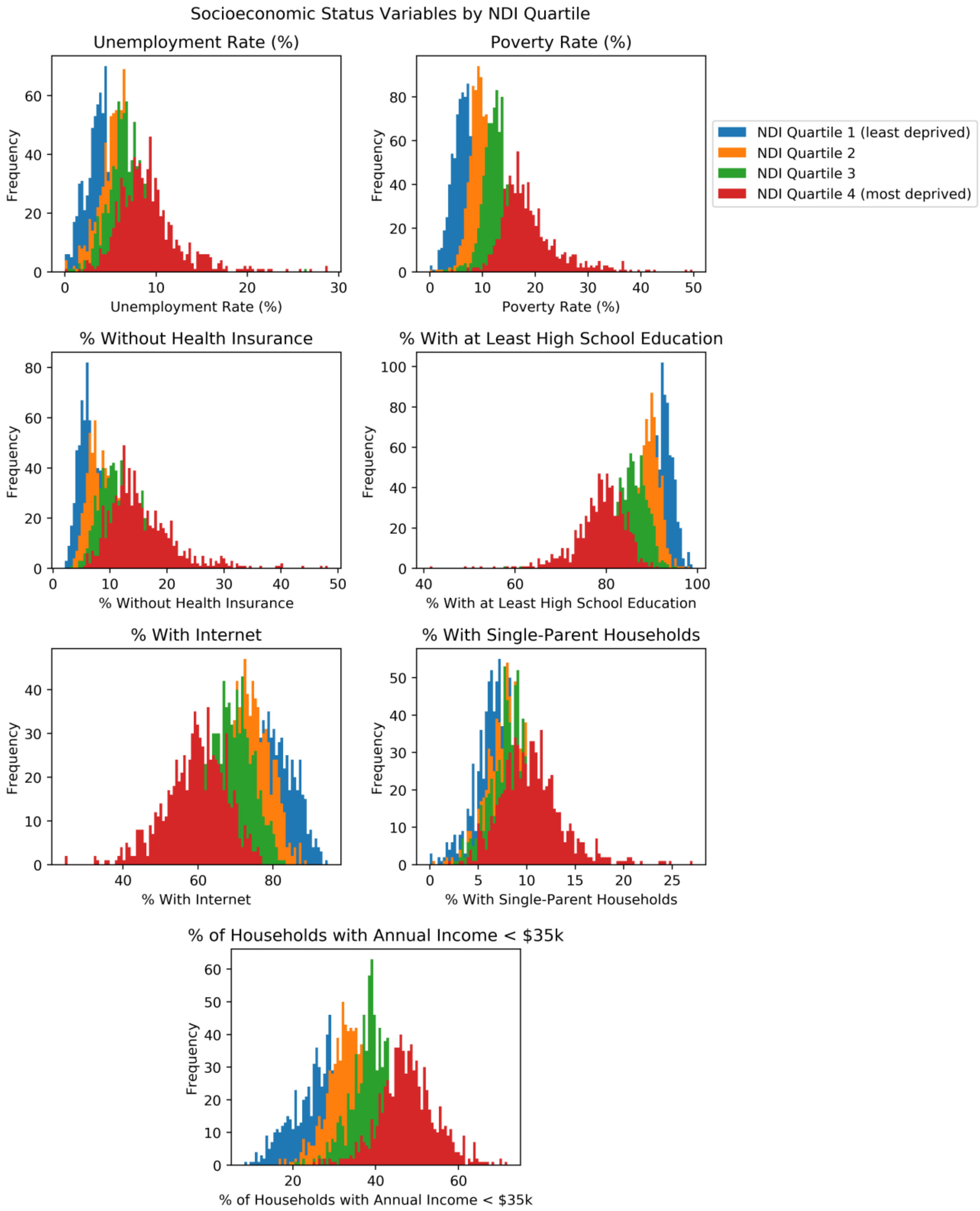

© 2020 Silver ER et al. *JAMA Network Open.*

eTable 1. Search Terms Used to Determine and Recode Cancer Type.

Cancer Type	Cancer Type-Recode
breast	breast
leukemia	blood
lung	lung
colon	gastrointestinal
lymphoma	blood
brain	brain
kidney	urologic
sarcoma	sarcoma
stomach	gastrointestinal
pancreatic	gastrointestinal
liver	gastrointestinal
ovarian	gynecologic
cervical	gynecologic
bone	bone
melanoma	melanoma
glioblastoma	brain
prostate	urologic
myeloma	blood
thyroid	thyroid
esophageal	gastrointestinal
uterine	gynecologic
throat cancer	head and neck
neuroblastoma	brain
uterus	gynecologic
tongue	head and neck
testicular	urologic
colorectal	gastrointestinal
bladder	urologic
astrocytoma	brain
renal cell	urologic
squamous cell	squamous_NOS
pancreas	gastrointestinal
breat	breast
medulloblastoma	brain
endometrial	gynecologic
rectal	gastrointestinal
skin cancer	melanoma
head and neck	head and neck
cervix	gynecologic
gallbladder	gastrointestinal
adenocarcinoma	adeno_NOS
gastric	gastrointestinal
retinoblastoma	eye
testicle	urologic
esophagus	gastrointestinal

© 2020 Silver ER et al. *JAMA Network Open.*

ductal carcinoma	breast
renal	urologic
bile duct	gastrointestinal
mastectomy	breast
neuroendocrine	neuroendocrine
peritoneal	peritoneal
blood cancer	blood
eye cancer	eye
intestinal	gastrointestinal
histiocytosis	histiocytosis
cerebral	brain
gastrointestin	gastrointestinal
appendix cancer	appendix
larynx	head and neck
luekemia	blood
ovaries	gynecologic
carcinoid	neuroendocrine
lukemia	blood
germ cell tumor	germ cell
rectum	gastrointestinal
laryngeal	head and neck
desmoplastic	sarcoma
wilms tumor	urologic
seminoma	urologic
ependymoma	brain
germ cell cancer	germ cell
thymoma	brain
urethra	urologic
urethral	urologic
adrenocortical	urologic
peritoneum	peritoneal
nueroblastoma	brain
lumpectomy	breast
myloma	blood
langerhans	histiocytosis
ductile carcinoma	breast
oligodendroglioma	brain
wilm's tumor	urologic
penile	urologic
ductal carcinoma in situ	breast
clear cell	clear_cell_NOS
mullerian	gynecologic
non-hodgkins lymphoma	blood
non hodgkins lymphoma	blood
neurblastoma	brain
neruoblastoma	brain
rhabdomyosaroma	sarcoma

© 2020 Silver ER et al. *JAMA Network Open.*

tyroid	thyroid
nuroblastoma	brain
nonhodgkins lymphoma	blood

© 2020 Silver ER et al. *JAMA Network Open.*

eTable 2. Search Terms and Regular Expressions (Regex) Used to Determine and Recode Mentions of Insurance, Out-of-Pocket Costs, and Treatment Type.

<table>
<tr><th>Insurance Type</th><th>Insurance Type-Recode</th></tr>
<tr><td>medi(.|)care</td><td>medicare</td></tr>
<tr><td>medi(.|)caid</td><td>medicaid</td></tr>
<tr><td>medi(.|)cade</td><td>medicaid</td></tr>
<tr><td>insurance</td><td>insurance</td></tr>
<tr><td>insurence</td><td>insurance</td></tr>
<tr><td>obama(.|)care</td><td>affordable care act</td></tr>
<tr><td>affordable care act</td><td>affordable care act</td></tr>
<tr><td>the aca</td><td>affordable care act</td></tr>
<tr><td>tri(.|)care</td><td>public health insurance</td></tr>
<tr><td>champus</td><td>public health insurance</td></tr>
<tr><td>champva</td><td>public health insurance</td></tr>
<tr><td>underinsure</td><td>underinsured</td></tr>
<tr><td>under(.|)insure</td><td>underinsured</td></tr>
<tr><td>under-insure</td><td>underinsured</td></tr>
<tr><td>no health ins.r.nce</td><td>uninsured</td></tr>
<tr><td>doesn(.|)t have ins.r.nce</td><td>uninsured</td></tr>
<tr><td>don(.|)t have health ins.r.nce</td><td>uninsured</td></tr>
<tr><td>no ins.r.nce</td><td>uninsured</td></tr>
<tr><td>doesn(.|)t have health ins.r.nce</td><td>uninsured</td></tr>
<tr><td>un.nsured</td><td>uninsured</td></tr>
<tr><td>don(.|)t have ins.r.nce</td><td>uninsured</td></tr>
<tr><td>not insured</td><td>uninsured</td></tr>
<tr><th>Out-of-Pocket Costs</th><th>Out-of-Pocket Costs-Recode</th></tr>
<tr><td>deduct.ble</td><td>deductible</td></tr>
<tr><td>transport</td><td>transportation</td></tr>
<tr><td>insur.nce premium</td><td>premium</td></tr>
<tr><td>out(.|)of(.|)pocket</td><td>out of pocket</td></tr>
<tr><td>co(.|)pay</td><td>co-pay</td></tr>
<tr><td>co(.|)payment</td><td>co-pay</td></tr>
<tr><td>transportation</td><td>transportation</td></tr>
<tr><td>medical bill</td><td>medical bills</td></tr>
<tr><td>hospital bill</td><td>hospital bills</td></tr>
<tr><td>debt</td><td>debt</td></tr>
<tr><td>lost wage</td><td>lost wages</td></tr>
<tr><td>isn(.|)t covered</td><td>not covered</td></tr>
<tr><td>won(.|)t cover</td><td>not covered</td></tr>
<tr><td>doesn(.|)t cover</td><td>not covered</td></tr>
<tr><td>not cover</td><td>not covered</td></tr>
</table>

© 2020 Silver ER et al. *JAMA Network Open.*

<table>
<tr><td>not covered</td><td>not covered</td></tr>
<tr><td>aren(.|)t covered</td><td>not covered</td></tr>
<tr><td>weren(.|)t covered</td><td>not covered</td></tr>
<tr><td>won(.|)t be covered</td><td>not covered</td></tr>
<tr><td>wasn(.|)t covered</td><td>not covered</td></tr>
<tr><td>isn(.|)t enough to cover</td><td>not covered</td></tr>
<tr><td>not enough to cover</td><td>not covered</td></tr>
<tr><td>not be covered</td><td>not covered</td></tr>
<tr><td>haven(.|)t been covered</td><td>not covered</td></tr>
<tr><td>not been covered</td><td>not covered</td></tr>
<tr><td>hasn(.|)t covered</td><td>not covered</td></tr>
<tr><td>didn(.|)t cover</td><td>not covered</td></tr>
<tr><td>isn(.|)t covered</td><td>not covered</td></tr>
<tr><td>won(.|)t cover</td><td>not covered</td></tr>
<tr><th>Treatment Type</th><th>Treatment Type-Recode</th></tr>
<tr><td>surgery</td><td>surgery</td></tr>
<tr><td>chemo</td><td>chemo</td></tr>
<tr><td>chemo(.|)therapy</td><td>chemo</td></tr>
<tr><td>immuno</td><td>immunotherapy</td></tr>
<tr><td>immuno(.|)therapy</td><td>immunotherapy</td></tr>
<tr><td>imuno(.|)therapy</td><td>immunotherapy</td></tr>
<tr><td>targeted therapy</td><td>targeted therapy</td></tr>
<tr><td>gene therapy</td><td>gene therapy</td></tr>
<tr><td>hysterectomy</td><td>surgery</td></tr>
<tr><td>oophorectomy</td><td>surgery</td></tr>
<tr><td>colectomy</td><td>surgery</td></tr>
<tr><td>radiation</td><td>radiation</td></tr>
<tr><td>hormone therapy</td><td>hormone therapy</td></tr>
<tr><td>mastectomy</td><td>surgery</td></tr>
<tr><td>lumpectomy</td><td>surgery</td></tr>
<tr><td>surgically removed</td><td>surgery</td></tr>
<tr><td>\w+[a-z]ectomy</td><td>surgery</td></tr>
</table>

© 2020 Silver ER et al. *JAMA Network Open.*

eAppendix 3. Keyword Searches for Deservingness Text Features.

Militaristic Metaphors

- battle
- battling
- battled
- fight
- fighting
- fought
- beat
- beating
- war against
- war with
- kick cancer(.|)s .ss
- kick cancer(.|)s butt
- warrior
- fighter
- war on

Bravery

- brave
- courag
- valiant
- valient
- strong
- strength
- hero

Self-Reliance

- hard(.|)working
- independ.nt
- self(.|)reli.n
- independ.nt
- stubborn
- proud
- determined
- resilli.nt
- resili.nt
- tenacity
- never ask for
- never asks for
- not ask for
- never asked for
- wouldn(.|)t ask for
- won(.|)t ask for

© 2020 Silver ER et al. *JAMA Network Open.*

- doesn(.|)t ask for
- don(.|)t ask for

Gratitude

- thank
- appreciate
- grateful
- greatful
- gratitude
- thanks
- appreciative
- apreciative
- apreciate
- your generosity

Warmth

- nicest
- kindest
- loving
- sweetest
- caring
- warmest

© 2020 Silver ER et al. *JAMA Network Open.*

eTable 3. Associations Between Amount Raised and Campaign Year. ANOVA: analysis of variance, SD: standard deviation.

Campaign Year	Mean (SD)	N	F-Statistic
2010	$4,927.00 ($4,032.68)	13	F(1, 143,572) = 191.26, *p* < 0.001
2011	$6,667.70 ($7,327.06)	115	
2012	$6,025.01 ($9,542.23)	682	
2013	$6,333.10 ($13,694.04)	2874	
2014	$5,973.91 ($10,613.32)	10366	
2015	$6,518.21 ($9,663.03)	19911	
2016	$8,267.85 ($12,106.13)	18061	
2017	$6,724.17 ($13,309.90)	29988	
2018	$6,004.44 ($11,998.87)	38837	
2019	$5,070.11 ($9,517.56)	22725	

eTable 4. Spearman Rank Correlations Between Amount Raised and Relevant Continuous Variables.

	Amount Raised	P-value
**Number of Contributors**	0.89	< 0.001
**Number of Social Media Shares**	0.54	< 0.001
**Goal Amount (Winsorized)**	0.48	< 0.001

© 2020 Silver ER et al. *JAMA Network Open.*

eTable 5. Descriptive Statistics for Continuous Variables.

	Mean (SD)	Median (Range)	N Missing (%)
**Amount Raised**	$6,367.77 ($11,611.49)	$3,165.00 ($0.00 - $1,010,060.00)	489.00 (0.34%)
**Goal Amount (Winsorized)**	$18,123.95 ($21,912.28)	$10,000.00 ($2,000.00 - $100,000.00)	3,826.00 (2.66%)
**Number of Contributors**	64.05 (129.89)	36.00 (0.00 - 12,300.00)	7,277.00 (5.05%)
**Average Contribution Amount**	$102.98 ($101.58)	$86.56 ($5.00 - $15,468.60)	7,284.00 (5.06%)
**Percent of Goal Raised**	43.99% (32.19%)	36.38% (0.00% - 100.00%)	4,315.00 (3.00%)
**Likes**	68.09 (593.18)	38.00 (0.00 - 209,300.00)	12,525.00 (8.69%)
**Shares**	436.71 (863.09)	250.00 (0.00 - 104,000.00)	8,750.00 (6.07%)

© 2020 Silver ER et al. *JAMA Network Open.*

eFigure 2. Log Amount Raised by Neighborhood Deprivation Index.

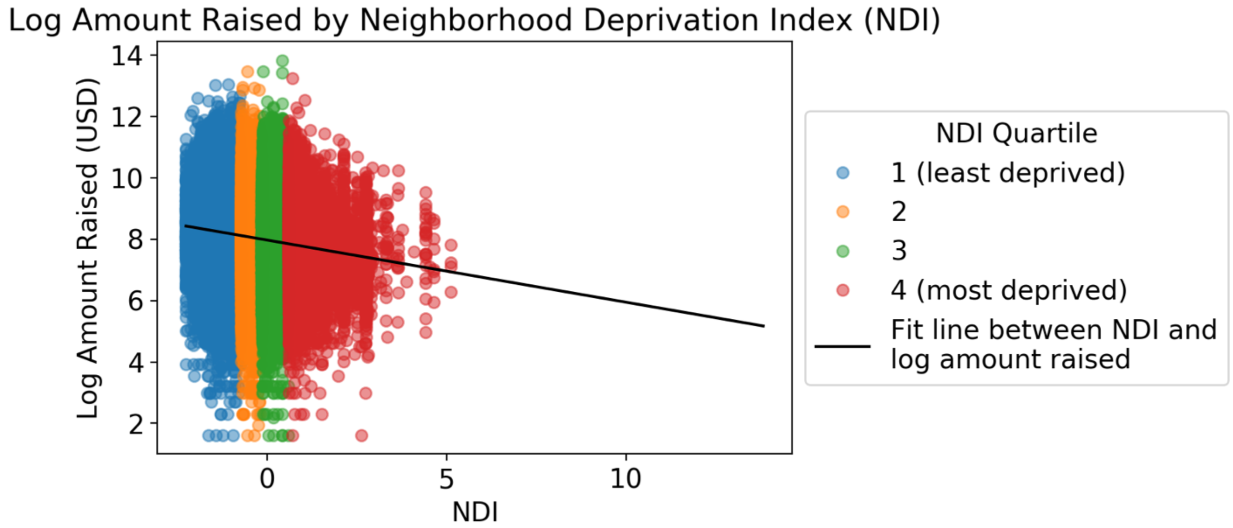

eTable 6. Expected and Observed Counts for Text Indicators by County Socioeconomic Status. NDI: Neighborhood Deprivation Index.

Variable	NDI Quartile			
	1 (Least Deprived)	2	3	4 (Most Deprived)
Bravery				
Percent Mentioned	30.23%	28.53%	27.83%	25.74%
Observed False	37156	30566	25702	9228
Observed True	16096	12203	9911	3199
Expected False	37945	30475	25376	8855
Expected True	15307	12294	10237	3572
Expected True - Observed True	789	-91	-326	-373
Militaristic Metaphors				
Percent Mentioned	58.38%	57.01%	57.03%	54.43%
Observed False	22165	18388	15302	5663
Observed True	31087	24381	20311	6764
Expected False	22740	18264	15208	5307

Expected True	30512	24505	20405	7120
Expected True - Observed True	575	-124	-94	-356
Cancer Type Mentioned				
Percent Mentioned	83.90%	83.51%	82.69%	81.50%
Observed False	8571	7052	6163	2299
Observed True	44681	35717	29450	10128
Expected False	8903	7150	5954	2078
Expected True	44349	35619	29659	10349
Expected True - Observed True	332	98	-209	-221
Insurance Mentioned				
Percent Mentioned	27.56%	28.20%	28.61%	28.16%
Observed False	38574	30708	25424	8927
Observed True	14678	12061	10189	3500
Expected False	38308	30767	25619	8940
Expected True	14944	12002	9994	3487
Expected True - Observed True	-266	59	195	13
Warmth				
Percent Mentioned	19.73%	18.10%	17.06%	14.79%
Observed False	42744	35029	29538	10589
Observed True	10508	7740	6075	1838
Expected False	43582	35002	29146	10170
Expected True	9670	7767	6467	2257

© 2020 Silver ER et al. *JAMA Network Open.*

Expected True - Observed True	838	-27	-392	-419
Out-of-Pocket Costs				
Percent Mentioned	41.81%	40.96%	39.72%	37.13%
Observed False	30988	25249	21468	7813
Observed True	22264	17520	14145	4614
Expected False	31612	25389	21141	7377
Expected True	21640	17380	14472	5050
Expected True - Observed True	624	140	-327	-436
Self-Reliance				
Percent Mentioned	15.79%	14.92%	14.16%	12.39%
Observed False	44841	36386	30569	10887
Observed True	8411	6383	5044	1540
Expected False	45350	36422	30328	10583
Expected True	7902	6347	5285	1844
Expected True - Observed True	509	36	-241	-304
Gratitude				
Percent Mentioned	72.12%	71.48%	71.43%	70.70%
Observed False	14845	12196	10173	3641
Observed True	38407	30573	25440	8786
Expected False	15102	12129	10100	3524
Expected True	38150	30640	25513	8903
Expected True -	257	-67	-73	-117

© 2020 Silver ER et al. *JAMA Network Open.*

Observed True				
Treatment Type				
Percent Mentioned	68.21%	67.49%	67.19%	64.44%
Observed False	16927	13905	11684	4419
Observed True	36325	28864	23929	8008
Expected False	17349	13934	11603	4049
Expected True	35903	28835	24010	8378
Expected True - Observed True	422	29	-81	-370

© 2020 Silver ER et al. *JAMA Network Open.*

eTable 7. Full Outputs From Multivariable Regression Model on Amount Raised (Log-Transformed). Coef: Beta coefficient, SE: standard error, CI: confidence interval, ref: reference value.

Variable	Coef. (SE)	Percent Difference in Expected Mean (95% CI)	z	P-Value
Intercept	7.614 (0.329)	NA	23.113	<0.001
NDI Quartile 2 (ref: NDI Quartile 1, Least Deprived)	-0.144 (0.006)	-13.39% (-14.44%, -12.28%)	-22.994	<0.001
NDI Quartile 3 (ref: NDI Quartile 1, Least Deprived)	-0.200 (0.007)	-18.14% (-19.18%, -17.06%)	-30.357	<0.001
NDI Quartile 4 (Most Deprived) (ref: NDI Quartile 1, Least Deprived)	-0.302 (0.010)	-26.07% (-27.46%, -24.65%)	-31.3	<0.001
Warmth is Mentioned (ref: Warmth Not Mentioned)	0.129 (0.007)	13.80% (12.30%, 15.26%)	19.549	<0.001
Gratitude is Mentioned (ref: Gratitude Not Mentioned)	0.001 (0.006)	0.09% (-1.00%, 1.21%)	0.156	0.876
Self-Reliance is Mentioned (ref: Self-Reliance Not Mentioned)	0.051 (0.007)	5.23% (3.77%, 6.72%)	7.096	<0.001
Cancer Type is Mentioned (ref: Cancer Type Not Mentioned)	0.091 (0.007)	9.58% (8.00%, 11.18%)	12.665	<0.001
Treatment Type is Mentioned (ref: Treatment Type Not Mentioned)	0.064 (0.006)	6.58% (5.44%, 7.79%)	11.188	<0.001
Insurance is Mentioned (ref: Insurance Not Mentioned)	0.014 (0.006)	1.39% (0.20%, 2.63%)	2.294	0.022
Out-of-Pocket Cost is Mentioned (ref: Out-of-Pocket Cost Not Mentioned)	0.071 (0.005)	7.36% (6.18%, 8.55%)	12.913	<0.001
Militaristic Metaphors (ref: Militaristic Metaphos Not Used)	0.112 (0.005)	11.87% (10.74%, 13.09%)	21.335	<0.001
Bravery is Mentioned (ref: Bravery Not Mentioned)	0.143 (0.006)	15.40% (14.11%, 16.65%)	24.801	<0.001

© 2020 Silver ER et al. *JAMA Network Open.*

Year				
2011 (ref: 2010)	0.118 (0.342)	12.48% (-42.48%, 119.90%)	0.344	0.731
2012 (ref: 2010)	0.088 (0.332)	9.23% (-43.05%, 109.38%)	0.266	0.79
2013 (ref: 2010)	0.019 (0.330)	1.94% (-46.58%, 94.64%)	0.058	0.953
2014 (ref: 2010)	-0.133 (0.329)	-12.41% (-54.07%, 67.03%)	-0.402	0.688
2015 (ref: 2010)	-0.048 (0.329)	-4.72% (-50.04%, 81.67%)	-0.147	0.883
2016 (ref: 2010)	0.124 (0.329)	13.26% (-40.61%, 115.98%)	0.378	0.705
2017 (ref: 2010)	-0.089 (0.329)	-8.54% (-52.05%, 74.37%)	-0.271	0.786
2018 (ref: 2010)	-0.202 (0.329)	-18.31% (-57.17%, 55.74%)	-0.614	0.539
2019 (ref: 2010)	-0.404 (0.329)	-33.20% (-64.97%, 27.38%)	-1.225	0.221
Goal Amount	0.000 (0.000)	0.00% (0.00%, 0.00%)	92.307	<0.001
Number of Shares	0.000 (0.000)	0.01% (0.01%, 0.00%)	29.405	<0.001
Number of Contributors	0.003 (0.000)	0.33% (0.30%, 0.30%)	138.168	<0.001

eReferences

1. Butler DC, Petterson S, Phillips RL, Bazemore AW. Measures of social deprivation that predict health care access and need within a rational area of primary care service delivery. *Health Serv Res*. 2013;48(2 Pt 1):539-559. doi:10.1111/j.1475-6773.2012.01449.x

2. Messer LC, Laraia BA, Kaufman JS, et al. The development of a standardized neighborhood deprivation index. *J Urban Health*. 2006;83(6):1041-1062. doi:10.1007/s11524-006-9094-x

© 2020 Silver ER et al. *JAMA Network Open.*
